# Editorial: Linking treatment target identification to biological mechanisms underlying mood disorders – Volume II

**DOI:** 10.3389/fpsyt.2024.1385955

**Published:** 2024-03-12

**Authors:** Shaohua Hu, Chee H. Ng, J. John Mann

**Affiliations:** ^1^ Department of Psychiatry, First Affiliated Hospital, Zhejiang University School of Medicine, Hangzhou, China; ^2^ Ministry of Education (MOE) Frontier Science Center for Brain Science & Brain-Machine Integration, Zhejiang, China; ^3^ Department of Psychology and Behavioral Sciences, Graduate School, Zhejiang University, Hangzhou, China; ^4^ The Key Laboratory of Precision Medicine for Mental Disorders, Hangzhou, China; ^5^ Department of Psychiatry, The Melbourne Clinic and St Vincent’s Hospital, University of Melbourne, Richmond, VIC, Australia; ^6^ Department of Psychiatry, Columbia University, New York, NY, United States; ^7^ Department of Radiology, Columbia University, New York, NY, United States

**Keywords:** mood disorder, major depressive diorder, biopolar disorder, brain-gut axis, inflammation

This issue of Frontier addresses the Research Topic: *Linking Treatment Target Identification to Biological Mechanisms Underlying Mood Disorders*.’ In the face of escalating global rates of mood disorders and their associated morbidity and mortality, improved treatment has never been more critical. Seeking treatment targets for medications identified by the molecular psychopathology of mood disorders is a huge advance over serendipity, which is how most antidepressant medications have been discovered to date. Encouragingly, progress in medical science is gradually illuminating the pathophysiology of mood disorders. For example, the microbiota-brain axis and its role in mediating immune imbalances present a promising frontier.

This Research Topic journal issue focuses on neuro-immune regulatory dysfunction related to mood disorders, treatments targeting the gut microbiota, new clinical research on therapeutic mechanisms, and anti-inflammatory treatment studies. These avenues hold the promise of transforming our approach to mood disorder treatments.

The Research Topic showcases four papers that delve into the relationship between treatment targets and the biological mechanisms underlying mood disorders. Li et al. dissect olfactory function in patients with bipolar disorder, offering a potential biomarker for early identification and providing nuanced insights into mood disorder subtypes and episodes. In another study, the interplay between inflammation, cytokines, and adolescent Major Depressive Disorder (MDD) was investigated; Maresin-1 was identified as a potential therapeutic target (Qiu et al.). Further, a review examines histamine’s role in MDD, introducing histamine receptors as potential targets for antidepressant therapy (Qian et al.). Another review surveys the epidemiology, pathogenic factors, clinical manifestations, and treatment landscape of pediatric and adolescent bipolar disorders (Liu et al.).

This Research Topic named Linking Treatment Target Identification to Biological Mechanisms Underlying Mood Disorders stands as a testament to collaborative efforts driving biological psychiatry research forward. From olfaction to inflammation resolution, from histamine to pediatric considerations, these papers collectively advance our understanding and beckon a future where the biological frontiers of mood disorders are explored and expanded. As we navigate these uncharted waters, one thing remains clear–the creation of knowledge-based treatment development is a central goal of psychiatric research.

We look forward to collaborating with our esteemed team of Topic Editors and editorial staff to enhance the scientific impact of our journal. Together, we aim to uphold the tradition of rigor and reproducibility while expanding our coverage to embrace emerging topics. Our Topic Editors Shaohua Hu (Zhejiang University School of Medicine, Hangzhou, China), Chee H Ng (The University of Melbourne Parkville, Australia), J. John Mann (Columbia University, New York City, United States), and Xiancang Ma (Department of Medical Imaging, First Affiliated Hospital of Xi’an Jiaotong University, Xi’an, China) will provide expertise in these relevant subject areas published by the journal, and I am confident in our ability to maintain the high scientific standards of the journal. We invite outstanding contributions and exciting research from our readers to continue making a significant impact on mental health worldwide.

## Author contributions

SH: Writing – original draft. CN: Writing – review & editing. JM: Writing – review & editing.

